# Triterpenoids from *Acacia ataxacantha* DC: antimicrobial and antioxidant activities

**DOI:** 10.1186/s12906-016-1266-y

**Published:** 2016-08-12

**Authors:** Abdou Madjid O. Amoussa, Latifou Lagnika, Mélanie Bourjot, Cathérine Vonthron-Senecheau, Ambaliou Sanni

**Affiliations:** 1 Unité de Biochimie et Biologie Moléculaire, Equipe de Biochimie et Substances Naturelles Bioactives, Faculté des Sciences et Techniques, Université d’Abomey-Calavi, Cotonou, 04 BP 0320 Bénin; 2 Laboratoire d’Innovation Thérapeutique, Faculté de Pharmacie, UMR CNRS-Unistra 7200, Illkirch, France

**Keywords:** *Acacia ataxacantha*, Triterpene, Betulinic acid-3-trans-caffeate, Antibacterial, Antifungal, Antioxidant

## Abstract

**Background:**

*Acacia ataxacantha* is a medicinal specie used extensively in traditional medicine of Benin republic to treat infectious diseases. Our previous study showed interesting antibacterial and antifungal activities against six strains of bacteria and six strains of fungi. The aim of this study was to investigate the antimicrobial and antioxidant activities of compounds isolated from *A. ataxacantha*.

**Methods:**

Chromatographic and spectroscopic methods were used to isolate and identify three compounds (**1**–**3**) from the bark of *A. ataxacantha.* Phytochemical investigation of *A. ataxacantha* (Fabaceae) led to the isolation of three triterpenoids (**1**–**3**). The structure of isolated compounds was established by differents spectroscopic methods such as UV, ^1^H NMR, ^13^C NMR, 2D NMR and Mass. All isolated compounds were tested for antimicrobial activity using agar disc-diffusion and microdilution methods. The radical scavenging activity of isolated compounds was assessed using 2,2-diphenyl-1-picrylhydrazyl (DPPH) method.

**Results:**

Phytochemical investigation led to the isolation and identification of lupeol (**1**), betulinic acid (**2**) and betulinic acid-3-trans-caffeate (**3**). Moderate antimicrobial activity was obtained with compound **3** against methicillin-resitant *Staphylococcus aureus*, *Enterococcus feacalis* and *Pseudomonas aeruginosa* with MIC value of 25 μg/ml and *Staphylococcus aureus* (MIC of 50 μg/ml). Compounds **3** was more active against *Staphylococcus epidermidis* and *Candida albicans* with a MIC value of 12.5 μg/ml in boths cases. Compounds **3** had also interesting antioxidant activity with an IC_50_ of 3.57 μg/ml compared to quercetin (1.04 μg/ml).

**Conclusion:**

The overall results of this study provide evidence that the compound **3**, isolated from *A. ataxacantha,* exhibit antimicrobial activity against Gram-positive and Gram-negative bacteria and yeast, especially against *C. albicans*.

## Background

Fabaceae, also known as Leguminosae represented by 730 genera like *Stylosanthes, Tamarindus, Caesalpinia, Acacia* and over 19400 species [1]. *Acacia* is a cosmopolitan genus containing in excess 1200 species and the highest density and the greatest diversity is found in tropical and subtropical regions, but also found throughout the world [2]. The aerial parts of different species of the genus *Acacia* are widely used in folk medicine due to their content of a variety of bioactive components which are responsible for numerous pharmacological properties such as hypoglycemic, anti-inflammatory, antibacterial, antihypertensive, analgesic, anticancer and antiatherosclerotic [3]. *Acacia ataxacantha* is widespread in much of sub-Saharan Africa. This species is a very thorny shrub with the height of 5 to 8 m. The leaves are alternate, with spine that carries 5 to 12 pairs of pinnae. On twigs, spines are short, clearly pointing down. The fruit pods are flattened, brownish red in the dry state. This specie has been reported in Benin, Nigerian and Kenya for its use in traditional medicine for the treatment of tooth decay, dysentery, bronchitis, cough and joint pain [4–6]. To the best of our knowledge, little phytochemical work has yet been done on *A. ataxacantha.* In a previous work, we reported the antioxidant, antifungal, antibacterial activities and toxicity of the bark extracts of this plant [7–9]. These results suggests that this plant might contain bioactive compounds that act as antimicrobial and antioxidant agents. It has been reported that in vitro tests do not necessarily confirm that the plant extracts are effective drugs or a suitable candidate for drug development, it provides a basis for understanding the effectiveness of the plant and leads in particular to the search for new active substances [10]. Therefore, the aim of this study was to isolate bioactive compound from *A. ataxacantha* barks and investigate their antimicrobial and antioxidant activities.

## Methods

### Plant material

*Acacia ataxacantha* barks were obtained from Ouidah, department of atlantic, South Bénin. Specimens were authenticated by Dr. Yedomohan, Botanist from National Herbarium of University of Abomey-Calavi. Voucher specimen (AA 6509/ HNB) have been deposited at the same Herbarium. The collected material was dried for four weeks in laboratory (22 °C), grinded into fine powder, and subjected to extraction.

### Extraction and isolation

Dry powdered bark of *A. ataxacantha* (250 g) was successively extracted three times (3 × 500 ml) for 72 h with hexane, dichloromethane, ethyl acetate and methanol by maceration at room temperature. The resulting extracts were filtered, concentrated under reduced pressure, and kept at 4 °C. The dichloromethane extract (2.5 g) was chromatographed by gradient elution on an open column (Silica gel Si 60, 0.063–0.200, mesh) using the mixture n-hexane/EtOAc and EtOAc-MeOH in increasing polarity to yield 36 fractions. These fractions were assembled into four fractions (A, B, C and D) according to the chromatographic profile obtained after thin layer chromatography (TLC) analysis. Fractions A (450 mg) and B (250 mg), soluble in dichloromethane, were recrystallized with methanol. The white precipitates obtained were purified by successive washing with methanol to obtain respectively compound **1** (25 mg) and **2** (32 mg). Purification of fraction C was done using preparative HPLC (Gilson VP 250/21, Nucleodur 100-5 C_18_ec, Macherey-Nagel, UV detection 220 and 254 nm) with a gradient elution 10:90 to 90:10 (solvent A: H_2_O + 0.1 trifluoroacetic acid (TFA), B: AcN + 0.1 TFA) to obtain 7 mg of compound **3**.

### Chemical elucidation of compounds

Structural determination of the isolated compounds was carried out by spectrophotometric methods (1D and 2D NMR, mass and UV spectrometry). 1D (^1^H, ^13^C) and 2D (COSY, NOESY, HSQC and HMBC) NMR spectrum were recorded at room temperature with a Bruker NMR spectrometer (400 MHz and 500 MHz), and mass spectra were recorded using LC-ESI-MS.

### Microbial strains

Bacterial cultures used in this study included *Staphylococcus aureus* (ATCC 6538), *Staphylococcus epidermidis* (CIP 8039), *Enterococcus faecalis* (ATCC 29212), Methicillin-Resistant *Staphylococcus aureus* and *Pseudomonas aeruginosa* (CIP 82118), obtained from Laboratoire de Biophotonique et Pharmacologie, University of Strasbourg, France. *Candida albicans* (CIP 4872) culture used in the present study was obtained from national laboratory of drug control in Cotonou (Bénin). Bacterial were maintained on Mueller-Hinton agar (MHA) and yeast on Sabouraud Dextrose Agar (SDA) at 4 °C. Sub-culturing was done weekly. The cells were inoculated in MH broth for bacteria (37 °C, 18 h) or SD broth for yeast (30 °C, 48 h) prior to the test.

### Bioautography and identification of antimicrobial compounds

This test was performed only on selected bacterial cultures which were remarkably inhibited by dichloromethane and ethyl acetate extracts, according to a modified version of the method of Srinivas et al., [11]. 10 μl of the extracts (20 mg/ml) were applied on a chromatographic plate (Pre-coated TLC-sheets ALUGRAM**®** silica gel 60 with fluorescent indicator UV_254_; layer thickness 0.20 mm for analytical TLC) followed by elution with a mixture of dichloromethane/methanol (98:2) and dried in air. The plates were run in duplicate. The first plate was used as the reference chromatogram. The spots in the chromatogram were visualized in UV chamber (wavelength 365 and 254 nm) and the plate was sprayed with sulfuric vanillin reagent. Other plates were used for the bioautography. The chromatograms were sprayed with bacterial culture (10^6^ CFU/ml) of *S. aureus*, Methicillin-resistant *S. Aureus*, *S. epidermidis*, *E. faecalis*, *P. aeruginosa* and fungi culture (2 × 10^5^ CFU/ml) of *C. albicans*. Each plate was incubated at (37 °C, 24 h) for bacteria and (30 °C, 48 h) for yeast. The inhibition zones were visualized by spraying the plates with *p*-iodonitrotetrazolium (INT, 2.0 mg/ml).

### Disc diffusion assay

The experiment was performed according to the method described by Qaralleh et al [12] with some modifications. For the determination of antimicrobial activity, cultures were adjusted to 10^6^ CFU/ml for bacteria and 2 × 10^5^ CFU/ml for yeast using 0.5 McFarland standards. Subsequently, cultures were inoculated into MHA for bacteria or SDA for yeast by spreading. The stock solutions of tested compounds were prepared by solubilizing 1 mg of compound in 50 μl of dimethyl sulfoxide 2.5 % (DMSO 2.5 %). Then, these solutions were diluted in 950 μl of Mueller-Hinton broth for bacteria and Sabouraud broth for yeast strain to obtain 1 mg/ml. The sterile discs of 6 mm of diameter were impregnated with 100 μg (50 μl, 2 mg/ml) of each compound. Discs of gentamicin (30 μg) and fluconazole (25 μg) were used as standard antibacterial and antifungal controls respectively. The plates were incubated at 37 °C, 24 h for bacteria and 30 °C, 48 h for yeast. The diameters of inhibitory zones (including the diameter of the discs) were measured after the incubation period and values superior to 7 mm (D ˃7 mm) were considered as active against microorganisms. All experiments were performed in triplicate and the antimicrobial activity was expressed as the mean of inhibition zone diameters.

### Minimum inhibitory concentration

The two-fold serial microdilution method was used to determine the minimum inhibitory concentration (MIC) values of isolated compounds against microorganisms [9]. 100 μl of isolated compound (100 μg/ml) and 50 μg/ml of antimicrobial standards (Gentamicin, Fluconazol) were serially diluted two-fold in triplicate with Mueller-Hinton broth for antibacterial test and Sabouraud broth for yeast test in 96-well microplates to make eight concentrations of isolated compound (0.78–100 μg/ml) and standards (0.39–50 μg/ml). 100 μl of fresh culture of bacteria (10^6^ CFU/ml) and yeast (2 × 10^5^ CFU/ml) were added to each well. DMSO (2.5 %) was used as negative control while gentamicin and fluconazole were used as positive controls.

### Minimum bactericidal and fungicidal concentration

The minimum bactericidal (MBC) and minimum fungicidal concentration (MFC) of isolated compounds was determined according to the method of Escalona-Arranz et al [13]. To determine the MBC and MFC, aliquots of 20 μl from all dilutions not showing any growth of bacteria and yeast were inoculated on sterile MHA plates (for bacteria) and SDA (for yeast) by spreading using swab sticks. Inoculated plates were incubated at 37 °C for 24 h for all bacteria, while those with yeast were incubated at 30 °C for 48 h. After incubation, the concentration at which there is no visible growth on the agar plate was recorded as the minimal bactericidal concentration (MBC) and minimal fungicidal concentration (MFC). The experiment was carried out in triplicate.

### Determination of MIC index

The MIC index (MBC/MIC) was calculated for each isolated compound and positive control drug to determine whether a compound had bactericidal/fungicidal (MBC/MIC ≤4) or bacteriostatic/fungistatic (4 < MBC/MIC <32) effect [14].

### In vitro antioxidant activity

The isolated compounds (2 mg) and quercetin (control) were dissolved in 1 ml of methanol HPLC-grade. Dilutions were performed to obtain a stock solution at 100 μg/ml. The antioxidant activity of isolated compounds on the stable radical 2,2-diphenyl-1-picrylhydrazyl (DPPH) was determined by the method developed by Danielle and Lall [15], with slight modifications. In this method 96-well plates were used. The stock solution (100 μl) of each isolated compound and quercetin was added separately to the wells in the top row. A two-fold serial dilutions was performed to obtain a concentration range from 1.56 to 100 μg/ml. Finally, 200 μl of methanolic solution of 2,2-diphenyl-1-picrylhydrazyl (2 %) were introduced in each well. The plates were allowed to develop in the dark for 30 min before the measurement of the absorbance at 517 nm using a Microplate Reader (Rayto-6500). The capability of each compound and the standard to scavenging the free radical was determined as inhibition percentage using the following formula:

$$ \mathrm{Inhibition}\ \mathrm{percentage}\ \left(\mathrm{I}\%\right) = \left[\left({\mathrm{A}}_{\mathrm{Blank}}\hbox{--}\ {\mathrm{A}}_{\mathrm{sample}}\right)/{\mathrm{A}}_{\mathrm{Blank}}\right]\kern0.5em \times \kern0.5em 100 $$

A_Blank_ is the absorbance of the control reaction (containing all reagents except the test sample) A_sample_ is the absorbance of sample/standard.

The concentration of compound reducing 50 % of free radical DPPH (IC_50_) was determined graphically. The assay was replicated three times and results are expressed as mean ± standard deviation.

### Statiscal analysis

All experiments were conducted in triplicate and the results were expressed as means ± standard deviation. The graph was performed using the Graph Pad Prism 6.1 software (Microsoft, USA).

## Results and discussion

### Phytochemical investigation

**Fig. 1:**
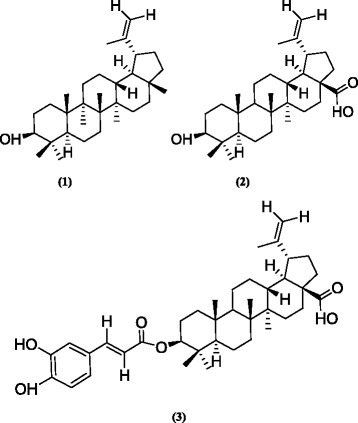
Isolated triterpenes from *Acacia ataxacantha*. (1): lupeol; (2): Betulinic acid; (3): Betulinic acid-3-trans-caffeate

The fractionation of the dichloromethane extract of *A. ataxacantha* using silica gel column chromatography led to the isolation of three compounds (**1**–**3**) identified as lupeol, betulinic acid and betulinic acid-3-trans-caffeate (Fig. 1).

1. White powder, ^1^H NMR (400 MHz, CDCl_3_): δ_H_ 0.72 (3H, s, H-24), 0.78 (3H, s, H-28), 0.81 (3H, s, H-25), 0.92 (3H, s, H-27), 0.97 (3H, s, H-23), 1.01 (3H, s, H-26), 1.66 (3H, s, H-30), 3.17 (1H, dd, J = 11Hz, 5 Hz, H-3), 4.55 (1H, dd, J = 2.4Hz, 1.4 Hz, H-29 α), 4.67 (1H, d, J = 2.4Hz, H-29 β). The ^1^H NMR spectrum of compound **1** revealed the presence of seven tertiary methyl protons at δ 0.72, 0.78, 0.81, 0.92, 0.97, 1.01 and 1.66 (integrated for 3H- each). A sextet of one proton at δ 2.37 assign to 19β-H is characteristic of lupeol. The H-3 proton showed a split doublet at δ 3.17 with a coupling constant of 11 Hz and 5 Hz while, a pair of doublet at δ 4.55 and δ 4.67 (1H, each) was indicative of olefinic protons at (H-29 a & b). Compound **1** was identified as lupeol. All spectral data were in agreement with literature [16–18].
2. White powder, ^1^H NMR (400 MHz, CDCl_3_): δ_H_ 0.65 (3H, s, H-24), 0.76 (3H, s, H-25), 0.86 (3H, s, H-26), 0.93 (3H, s, H-23), 0.97 (3H, s, H-27), 1.65 (3H, s, H-30), 3.32 (1H, m, H-3), 4.56 (1H, s, H-29 α). The ^1^H-NMR spectrum of compound 2 exhibited signals for methyl groups at δ 0.65, 0.76, 0.86, and 0.93, 0.97 and 1.65. The ^1^H NMR spectrum also displayed signals for olefinic hydrogens at δ 4.69, δ 4.56 (2H, s) for H-29 and hydrogen attached to carbon bearing OH (H-3) at δ 3.32 (1H, s), respectively. The spectral data of compounds **2** were in agreement with previously published data of betulinic acid [19, 20].
3. White amorphous powder. The molecular formula of **3** determined to be C_39_H_54_O_6_ by positive mode LC-ESI-MS data at m/z 641.38035 [M + Na]^+^ (calcd for C_39_H_54_O_6_, 641.38035). The UV spectrum exhibited absorption maxima at 245 and 320 nm, suggesting the presence of an aromatic ring in the molecule. ^1^H and ^13^C NMR spectral data were resumed in Table 1. The 2D experiments (COSY, NOESY, HSQC and HMBC) were performed using standard Bruker programs.

**Table 1:** NMR Spectroscopic Data (500 MHz, CDCl_3_) of compound **3** (betulinic acid-3-trans-caffeate)

Position	Compound 3	
	δ_H_ (*J* in Hz)	δ_C,_ type
1	1.73 (m), 1.07 (m)	39.2, CH_2_
2	1.68 (m), 1.68 (m)	24.6, CH_2_
3	4.57 (t, 5.1)	81.2, CH
4	–	38.3, C
5	0.91 (m)	55.7, CH
6	1.53 (m), 1.46 (m)	19, CH_2_
7	1.39 (m), 1.51 (m)	35.1, CH_2_
8	–	42.7, C
9	1.43 (m)	50.7, CH
10	–	34.6, C
11	1.12 (m), 1.46 (m)	21.8, CH_2_
12	1.26 (m), 1.74 (m)	26.4, CH_2_
13	2.39 (m)	39.0, CH
14	–	43.3, C
15	1.59 (m), 1.20 (m)	30.5, CH_2_
16	2.26 (m) 1.44 (m)	32.9, CH_2_
17	–	56.8, C
18	1.59(m)	49.5, CH
19	3.0(m)	47.1, CH
20	–	150, C
21	1.93 (m), 1.37 (m)	31.4, CH_2_
22	1.50 (m), 1.92 (m)	37.9, CH_2_
23	0.86 (s)	28.3, CH_3_
24	0.89 (s)	16.3, CH_3_
25	0.93 (s)	16.9, CH_3_
26	0.97 (s)	16.4, CH_3_
27	0.97 (s)	14.9, CH_3_
28	–	177.8, C
29	4.60, 4.57 (brs)	110, CH_2_
30	1.68 (s)	19.6, CH_3_
1′	–	128, C
2′	7.07 (d, 1.2)	122.6, CH
3′	–	115.8, C
4′	–	146.1, C
5′	6.86 (d, 8.0)	143.9, CH
6′	6.99 (dd, 8.0, 1.2)	114.6, CH
7′	7.53 (d, 15.9)	144.3, CH
8′	6.24 (d, 15.9)	116.9, CH
9′	–	167.7, C

Assignments were based on 2D NMR including HSQC, HMBC and NOESY. Well-resolved couplings are expressed with coupling patterns and coupling constants in hertz in parentheses

The compound **3** was inferred to be a triterpene, based on the ^1^H NMR spectrum with a broad range of aliphatic signals including six methyl singlets and the ^13^C NMR spectrum with 30 carbons (Table 1). Further investigation of ^13^C NMR spectrum revealed the characteristic signals for carboxylic acid (δ_C_ 177.8), vinyl carbons (quaternary C at δ_C_ 150.0 and CH_2_ at δ_C_ 110.0) as well as an oxygen-bearing methine (δ_C_ 81.2). This information suggested that the compound **3** is a derivative of betulinic acid bearing an additional aromatic ester moiety at C-3. The aromatic ester was identfied as caffeate by ^1^H NMR displaying *trans* olefin signal at δ 6.24 (d, *J* = 15.9 Hz). The compound **3** was identified as betulinic acid-3-trans-caffeate, which was confirmed by comparing its data to literature values [21–23].

### Antimicrobial activity

**Fig. 2:**
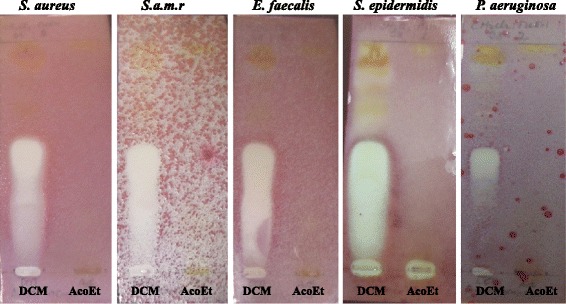
Bioautography of *A. ataxacantha* extracts. DCM: dichloromethane; AcoEt: Ethyle acetate; *S.aureus*: *Staphylococcus aureus*; *S.am.r*: *Staphylococcus aureus* meticilline resistent; *S. epidermidis*: *Staphylococcus epidermidis; E. feacalis: Enterococcus feacalis; P. aeruginosa*: *Pseudomonas aeruginosa*. *White* areas indicate where reduction of INT to the coloured formazan did not take place due to the presence of compounds that inhibited the bacterial growth

**Fig. 3:**
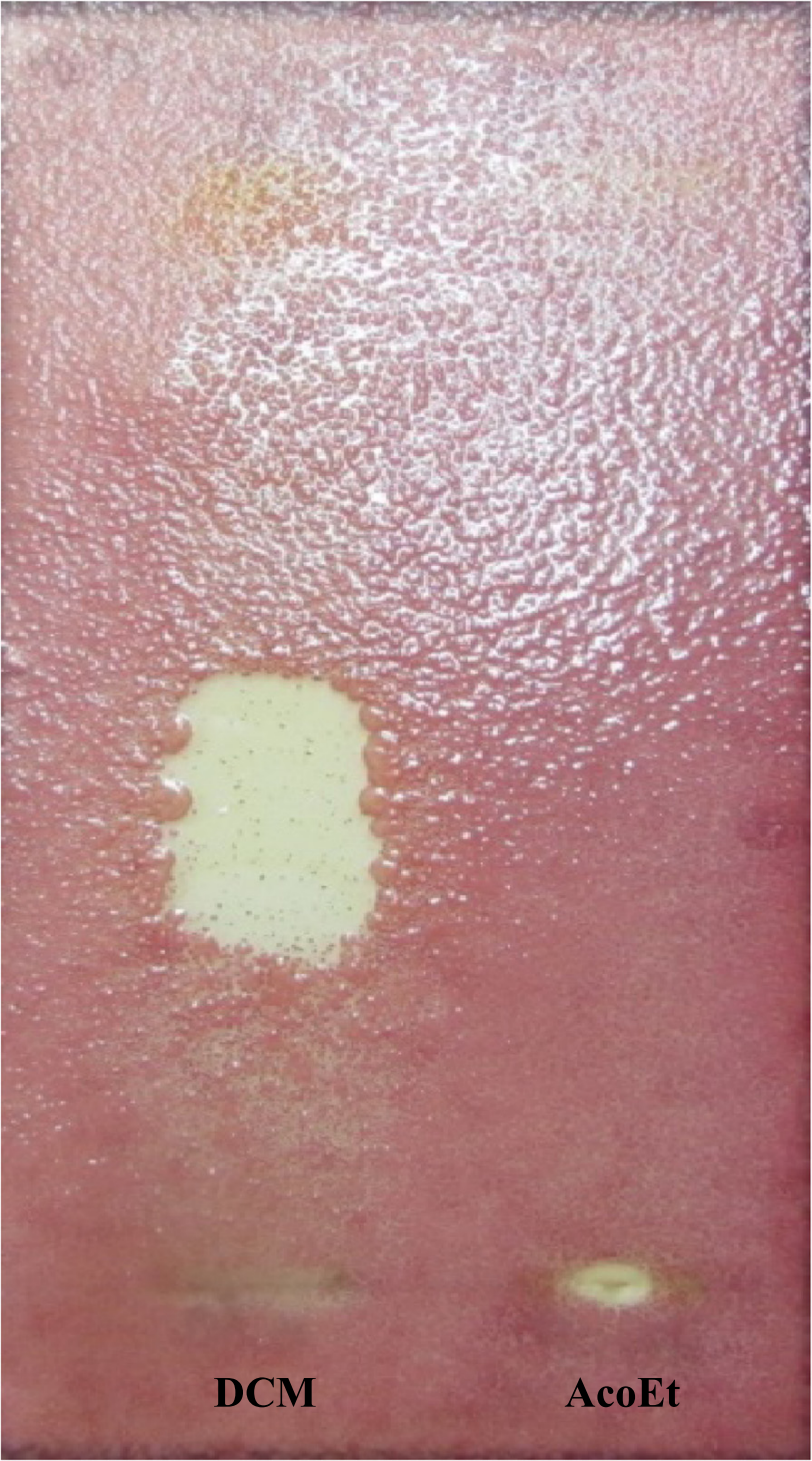
Bioautography of *A. ataxacantha* extracts sprayed with *Candida albicans* inoculum. DCM: dichloromethane; AcoEt: Ethyle acetate. *White* areas indicate where reduction of INT to the coloured formazan did not take place due to the presence of compounds that inhibited the bacterial growth

The bioautography technique has been used to identify the bioactive constituents from *A. ataxacantha* extracts. Inhibition zones of antimicrobial components were observed as white spots on a purple red background (Fig. 2). These white areas indicate the presence of antimicrobial compounds which inhibit the growth of microorganisms which did not support the reduction of INT to the coloured formazan [24]. In a previous study, dichloromethane (DCM) extract demonstrated the lowest MIC against *S. aureus*, methicillin-resistent *S. aureus*, *S. epidermidis*, *E. faecalis* and *P. aeruginosa* [9]. Hence, these bacteria were selected for the bioautography assay to identify antimicrobial compounds. The bioautography assay exhibited inhibition zones (Rf 0.59) for dichloromethane extract against both Gram positive (*S. aureus*, methicillin-resistent *S. aureus*, *S. epidermidis* and *E. faecalis*) and Gram negative (*P. aeruginosa*) (Fig. 2). Interestingly, the bioautogram with *C. albicans* showed inhibition zones at Rf 0.59 (Fig. 3) indicating the same compound was responsible for the antifungal activity against *C. albicans*.

Subsequently, an experiment was conducted to isolate and identify the actives compounds. The isolated compounds were investigated for their antimicrobial activity against Gram-positive (*S. aureus*, methicillin-resistent *S. aureus*, *S. epidermidis* and *E. faecalis*), Gram-negative (*P. aeruginosa*) and yeast (*C. albicans*). The results obtained with disc diffusion assay were presented in Table 2. Only Compound **3** out of the isolated compounds was active against all tested microorganisms (Table 2). The diameter of inhibition of compound **3** ranged from 15.7 to 23.3 mm. The highest inhibitory effect was observed against *S. epidermidis* with inhibition diameter of 23.3 mm. Overall, the Gram-positive bacteria showed a greater susceptibility to compound **3** while *P. aeruginosa* (Gram-negative) showed moderate sensitivity. Compounds **1** and **2** did not inhibit the growth of microorganisms at 100 μg/disc. Previous study showed that compounds **1** and **2** were active against *Escherichia Coli, Bacillus Subtilis and Staphylococcus aureus* with MIC values ranged from 100 to 200 μg/ml [25]. The negligible antimicrobial activity of compounds **1** and **2** could be explained by the concentration used. However, it was reported that betulinic acid was not active against the Gram-positive, Gram-negative bacteria and yeast [26].

**Table 2:** Preliminary antimicrobial testing of isolated compounds (**1**–**3**) by determination the zone of inhibitory (mm)

Bacteria/Fungus	1 (100 μg.disc^-1^)	2 (100 μg.disc^-1^)	3 (100 μg.disc^-1^)	Gentamicin(30 μg.disc^-1^)	Fluconazole (25 μg.disc^-1^)
*S. a*	NA	NA	18.3 ± 0.1	22.5 ± 0.0	Nt
*S.a*.m.r	NA	NA	20.0 ± 0.1	18.5 ± 0.0	Nt
*S. ep*	NA	NA	23.3 ± 0.0	20.5 ± 0.0	Nt
*E. f*	NA	NA	21.0 ± 0.1	22.0 ± 0.0	Nt
*P. a*	NA	NA	15.3 ± 0.0	14.0 ± 0.1	Nt
*C. a*	NA	NA	15.7 ± 0.0	NT	13.7 ± 0.1

Each value is expressed as means (*n* = 3) ± standard deviation (SD)

*S.a :Staphylococcus aureus;Samr: Staphylococcus aureus* methicillin resistant*; S.ep :Staphylococcus epidermidis*; *E.f: Enterococcus faecalis; P. a: Pseudomonas aeruginosa; C. a: Candida albicans.* **1**: lupeol; **2**: betulinic acid; **3**: betulinic acid-3-trans-caffeate. NA: not actif; NT: not tested

The present study has also assessed the quantitative antimicrobial activity of isolated compounds by determining their MIC, MBC and MFC. The results were reported in Table 3. Many reports consider the antimicrobial activities of compounds to be significant if the MIC is 10 μg/ml or lower, moderate if 10 ˂ MIC ≤ 100 μg/ml and low if MIC ˃ 100 μg/ml [27, 28]. Referring to these criteria, the tested compounds had moderate antimicrobial activity with MICs ranged from 12.5 to 50 μg/ml. MBC and MFC values varied between 25 to 50 μg/ml. Only compound **3** showed antimicrobial activity against tested microorganisms at different level. This compound was active against *S. aureus* and *P. aeruginosa* (MIC and MBC were 25 μg/ml in both cases), methicillin-resitant *S. aureus* and *E. faecalis* (MIC or MBC were 50 μg/ml in both cases). The lowest MIC (12.5 μg/ml) of compound **3** was recorded against *S. epidermidis* with a MBC value of 25 μg/ml. Aba et al., also demonstrated the antimicrobial activity of the amyrenol, a triterpene isolated from the roots of *A. ataxacantha* with MIC value of 12 μg/ml and MBC/MFC of 25 μg/ml against *Bacillus subtilis, Escherichia coli and Salmonella typhi* [29]. Our results and those obtained by Aba et al, demonstrated the importance of triterpenes in the antimicrobial activity of *A. ataxacantha*.

**Table 3:** Minimum inhibitory concentration (MIC) and Minimum bactericidal and fungicidal concentrations (MBC, MFC) of compound 3 from *A. ataxacantha*

**Minimum inhibitory concentrations (μg/ml)**						
Microorganisms^a^	Gram (+) bacteria				Gram (-) bacteria	Yeast
	*S. a*	*S.a.m.r*	*S. ep*	*E. f*	*P. a*	*C. a*
**3** ^b^	50	25	12.5	25	25	12.5
Gentamicin	0.39	0.39	0.78	0.39	0.78	Nt
Fluconazole	Nt	Nt	Nt	Nt	Nt	0.78
**Minimum bactericidal and fungicidal (μg/ml)**						
**3** ^b^	50	50	50	50	25	25
Gentamicin	0.78	1.56	1.56	0.78	1.56	Nt
Fluconazole	Nt	Nt	Nt	Nt	Nt	1.56
**MIC index**						
**3** ^b^	1	2	4	2	1	2
Gentamicin	2	4	2	2	2	Nt
Fluconazole	Nt	Nt	Nt	Nt	Nt	2

^a^ *S.a : Staphylococcus aureus; S.a.m.r : Staphylococcus aureus* methicillin resitant*; S.ep : Staphylococcus epidermidis*; *E.f: Enterococcus faecalis; P.a: Pseudomonas aeruginosa; C.a: Candida albicans*

^b^ **3**: betulinic acid-3-trans-caffeate

Nt: not tested. MIC, MBC or MFC of compounds **1** and **2** were not determined

The mechanism of antibiosis of the compound **3** was calculated using MIC index as described by Stefanovic and Comic [14], to elucidate whether the observed antibacterial effect was bactericidal or bacteriostatic. Higher values of MBC and MFC than those of MIC indicates the bacteriostatic or fungistatic nature of the compound **3** against methicillin-resistant *S. aureus*, *S. epidermidis*, *E. faecalis* and *C. albicans*. The same values of MBC and MIC observed against *S. aureus* (50 μg/ml) and *P. aeruginosa* (25 μg/ml) indicated the bactericidal nature of compound **3**. MIC and MBC values of compound **3** were most interesting against *P. aeruginosa* which is a Gram-negative bacterium. Several decades ago, the search for antimicrobials was still focused on the discovery of natural compounds able to inhibit Gram-negative bacteria, which are dangerous and causing infectious diseases. The Gram-negative cell wall (made up of lipopolysaccharide) is complex and multilayered structure, which makes access to membrane more restricted and barrier to many environmental substances including synthetic and natural antibiotics [30]. The results of this study indicate that the compound **3**, isolated from the bark of *A. ataxacantha*, could be an agent able to cross this complex barrier. In this study, compound **3** (betulinic acid-3-trans-caffeate) is found more active than compound **2** (betulinic acid). This observation is in accordance with the structure-activity relationship as reported previously [20, 31].

### Antioxidant activity

The antioxidant activity of isolated compounds (**1**–**3**) were determined using DPPH method and the results are reported in Table 4. Compounds **3** (betulinic acid-3-trans-caffeate) had significant antioxidant activity with an IC_50_ of 3.57 μg/ml compared to quercetin (control) 1.04 μg/ml. Compound **1** (lupeol) showed moderate activity with an IC_50_ value of 16.77 μg/ml while compound **2** (betulinic acid) had weak DPPH scavenging activity with an IC_50_ of 25.15 μg/ml. The antioxidant activity of lupeol was previously reported [32, 33]. The interesting antioxidant activity of compound **3** could be attributed to the phenolic nature of the caffeate substituent. The antioxidant activity of Alkyl caffeates have been also reported [34].

**Table 4:** Antioxidant activity of compounds (**1**–**3**) isolated from *A. ataxacantha*

Samples	IC_50_ (μg/ml)
Compounds	
**1**	16.77 ± 0.18
**2**	25.15 ± 0.01
**3**	3.57 ± 0.02
Reference	
Qercetin	1.04 ± 0.01

The value of IC_50_ are expressed as means (*n* = 3) ± standard deviation (SD)

**1**: lupeol, **2**: betulinic acid, **3**: betulinic acid-3-trans-caffeate

## Conclusion

The results of the present study showed that the betulinic acid-3-trans-caffeate isolated from the bark of *A. ataxacantha* possesses good antimicrobial and antioxidant potency. This compound could be a candidate for structure-activity study in the case of the development of novel antimicrobial agents with an improved therapeutic index. This is the first report on the presence of lupeol, betulinic acid and betulinic acid-3-trans-caffeate in this specie. Further studies are in progress to identify the synergy between the isolated compounds and standard antibiotics.

## Abbreviations

1D, dimensional; 2D, two dimensional; ATCC, American Type Culture Collection; CFU, Colony-forming unit; CIP, collection institute Pasteur; COSY, Correlation Spectroscopy; DCM, dichloromethane; DMSO, Dimethyl sulfoxide; DPPH, 2,2-diphenyl-1-picrylhydrazyl; EtoAc, ethyl acetate; HMBC, Heteronuclear Multiple Bond Correlation; HPLC, High performance liquid chromatography; HSQC, Heteronuclear single quantum coherence spectroscopy; INT, *p*-iodonitrotetrazolium; MBC, minimum bactericidal concentration; MeOH, methanol; MFC, minimum fungicidal concentration; MHA, Mueller-Hinton agar; MIC, minimum inhibitory concentration; NMR, Nuclear magnetic resonance; NOESY, Nuclear Overhauser Effect Spectroscopy; SDA Sabouraud Dextrose Agar; TFA, trifluoroacetic acid; TLC, thin layer chromatography
